# Driving Wi‐Fi IoT Sensors by a Hybrid Magneto‐Mechano‐Electric Energy Generator Extracting a Power of over 50 mW

**DOI:** 10.1002/advs.202405526

**Published:** 2024-09-30

**Authors:** Seungah Lee, Chang Min Baek, Gang Hyeon Kim, Srinivas Pattipaka, Hyunseok Song, Jongmoon Jang, Geon‐Tae Hwang, Jungho Ryu

**Affiliations:** ^1^ School of Materials Science and Engineering Yeungnam University Gyeongsan 38541 South Korea; ^2^ Department of Materials Science and Engineering Pukyong National University Busan 48513 South Korea; ^3^ Department of Electronic Engineering Yeungnam University Gyeongsan 38541 South Korea; ^4^ Institute of Materials Technology Yeungnam University Gyeongsan 38541 South Korea

**Keywords:** energy harvester, IoT, magnetic flux, magneto‐mechano‐electric, Wi‐Fi

## Abstract

Energy harvesting technology is mainly used as a power source for driving Internet of Things (IoT) devices. However, the output power of conventional harvesting devices are limited, suitable only for low‐power‐consumption IoT sensors based on Bluetooth communication. In contrast to Bluetooth, wireless fidelity (Wi‐Fi) communication offers superior real‐time monitoring and transmission capabilities, but requires more power in the range of hundreds of milliwatts or higher. Therefore, the hybridization of three energy conversion devices, namely, piezoelectric magneto‐mechano‐electric (MME) generator, electromagnetic (EM) induction coil, and triboelectric nanogenerator (TENG) is proposed as a standalone power source for Wi‐Fi communication sensors. By integrating these three mechanisms, the hybrid MME energy harvester can achieve an output power exceeding 50 mW at the second harmonic resonance condition under the alternating current (AC) magnetic field of 10 Oe. Furthermore, it can successfully drive the Wi‐Fi sensor, enabling continuous real‐time monitoring without the degradation of charged power in a supercapacitor. These results highlight that energy harvesting technology is not limited to low‐power devices but can also be applied to Wi‐Fi communication sensors and beyond.

## Graphical abstract

The hybridization of three energy conversion devices, piezoelectric magneto‐mechano‐electric (MME) generator, electromagnetic (EM) induction coil, and triboelectric nanogenerator (TENG) is introduced as a standalone power source for Wi‐Fi communication sensors. The hybrid generator extracts over 50 mW and can power a Wi‐Fi sensor for continuous real‐time monitoring, demonstrating its potential beyond low‐power devices.

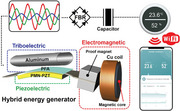

## 1 Introduction

Energy harvesting technology, which captures usable electrical energy from various ambient energy sources, has emerged as a sustainable, maintenance‐free, and autonomous power solution for Internet of Thing (IoT) systems in the fourth industrial era.^[^ ^1^, ^2^, ^3^, ^4^, ^5^, ^6^, ^7^, ^8^, ^9^ ^]^ Several energy conversion mechanisms, including piezoelectric, triboelectric, electromagnetic (EM), and magnetoelectric (ME) mechanisms, have been developed and employed for harvesting vibrational, mechanical, or magnetic energy in diverse IoT applications. Nevertheless, the power output obtained from these energy conversion mechanisms typically remains limited to a few milliwatts or less; thus, they are primarily suitable for powering ultralow‐power IoT devices utilizing Bluetooth low‐energy (BLE) communication.^[^ ^10^, ^11^, ^12^ ^]^

In contrast to BLE, wireless fidelity (Wi‐Fi) communication offers several advantages, such as efficient transmission, real‐time data transfer with minimal delay, high data transfer speed, scalability, reduced interference, integration with cloud services, and device‐to‐device communication. Nonetheless, Wi‐Fi communication devices generally consume tens to hundreds of milliwatts of power; thus, the energy harvesting system must generate corresponding power levels for its utilization as a self‐power source for Wi‐Fi IoT sensors. Achieving power outputs in the range of several tens of milliwatts can enable the integration of energy‐harvesting devices into a broader range of Wi‐Fi‐based mobile or small‐scale electronic devices, such as smartphones, wearable technology, and multifunctional IoT sensors.^[^ ^13^, ^14^ ^]^

To date, studies have focused on the development of various energy conversion mechanisms aimed at harvesting vibrational, mechanical, or magnetic energy to enhance the output power.^[^ ^9^, ^11^, ^12^, ^15^, ^16^, ^17^, ^18^, ^19^, ^20^, ^21^, ^22^ ^]^ For instance, piezoelectric magneto‐mechano‐electric (MME) generators,^[^ ^3^, ^5^, ^16^, ^23^, ^24^, ^25^, ^26^ ^]^ EM induction generators,^[^ ^27^, ^28^, ^29^, ^30^, ^31^, ^32^, ^33^, ^34^ ^]^ and magneto‐mechano‐triboelectric nanogenerators (MMTENGs)^[^ ^5^ ^]^ have extensively been developed as magnetic energy harvesting technologies. The piezoelectric MME generator is recognized for its effectiveness in capturing low‐frequency magnetic energy, such as the stray magnetic fields surrounding current‐carrying cables, boasting an energy conversion efficiency of ≈20% despite its compact size. However, owing to its fundamental reliance on piezoelectric energy conversion, the MME generator typically yields a relatively small generated current, although it produces a significantly higher output voltage.^[^ ^22^ ^]^ Conversely, the EM induction effect represents a well‐established energy conversion mechanism capable of generating high current output but low voltage.^[^ ^9^, ^16^, ^35^ ^]^ The energy conversion efficiency of an EM generator tends to decrease with decreasing frequency and device volume as it depends on the number of wound coils in the device. To overcome this limitation, the concept of hybrid energy harvesting has emerged, wherein multiple energy conversion mechanisms are simultaneously utilized. Recently, hybrid harvesting technology, with the combination of MME generators and EM induction coils, has been developed, aiming to improve the harvested output efficiency and power.^[^ ^12^, ^20^, ^35^, ^36^, ^37^ ^]^

**Figure 1:**
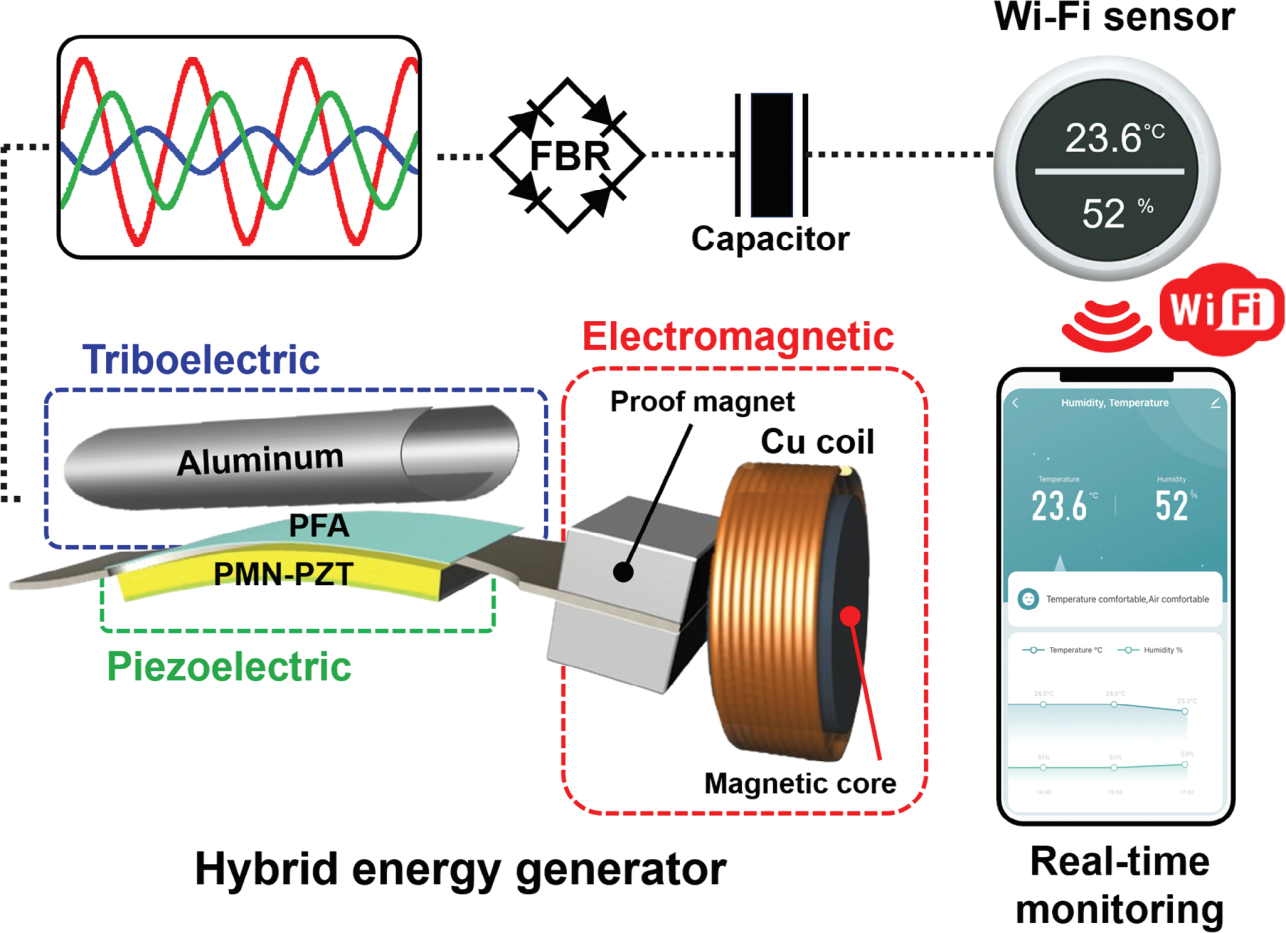
Structural design of the hybrid energy generator, and schematic of operation of the Wi‐Fi communication system by the integration of the MME generator, EM coil, and TENG. The electric power generated from each energy harvesting device is rectified and saved to the supercapacitor. The charged energy is sufficient for driving multifunctional sensors with smart mobile devices through Wi‐Fi communication.

In this study, we investigated a novel hybrid energy harvesting system, wherein the MME generator hybridized with a magnetic flux concentrated EM induction coil is integrated with a triboelectric nanogenerator (TENG), operating at the second harmonic resonance condition of a piezoelectric cantilever beam, as shown in **Figure** **1**. This hybridization is aimed at improving the output power of the magnetic energy harvesting system. In particular, we introduced a copper (Cu) solenoid coil with a magnetic flux‐concentrating core to depict the EM induction part of the hybrid MME generator. The magnets positioned at the end of the cantilever structure were vibrated by an applied external alternating current (AC) magnetic field in the second harmonic bending mode. This vibration altered the net magnetic field within the solenoid, thus generating an EM induction current in addition to the energy produced by the piezoelectric component of the cantilever beam. Moreover, to further enhance the harvesting performance, we also incorporated TENG into the MME system, thereby combining multiple energy conversion mechanisms.^[^ ^5^, ^18^, ^21^, ^38^, ^39^, ^40^, ^41^ ^]^

As each potential energy conversion mechanism presents distinct advantages and challenges, the hybridization of these energy conversion mechanisms must be optimized within an integrated single MME device to achieve compactness. To enhance the performance of the piezoelectric MME generator, the design of the geometry and material of the elastic constituent, along with the location of the piezoelectric material in the cantilever beam, were optimized to efficiently utilize the second harmonic bending mode rather than the conventional first harmonic bending mode. Furthermore, to simultaneously improve the output power from both piezoelectric MME energy conversion and the EM induction part, a high‐permeability magnetic core was incorporated into the Cu solenoid coil to achieve a concentration effect of the external magnetic field. Finally, for the hybridization of the high‐performance TENG in the hybrid MME generator, the triboelectric materials and integrating locations of the TENG part were carefully optimized.

By integrating these three energy conversion mechanisms and leveraging the magnetic flux concentration effect, the hybrid energy harvesting system can generate an output power exceeding 50 mW under an AC magnetic field of 10 Oe. The output power is sufficient for the subsequent transmission and real‐time Wi‐Fi communication with both a multifunctional sensor and a smart mobile device.

## 2 Result and Discussion

### 2.1 Optimization of MME Energy Generator

**Figure 2:**
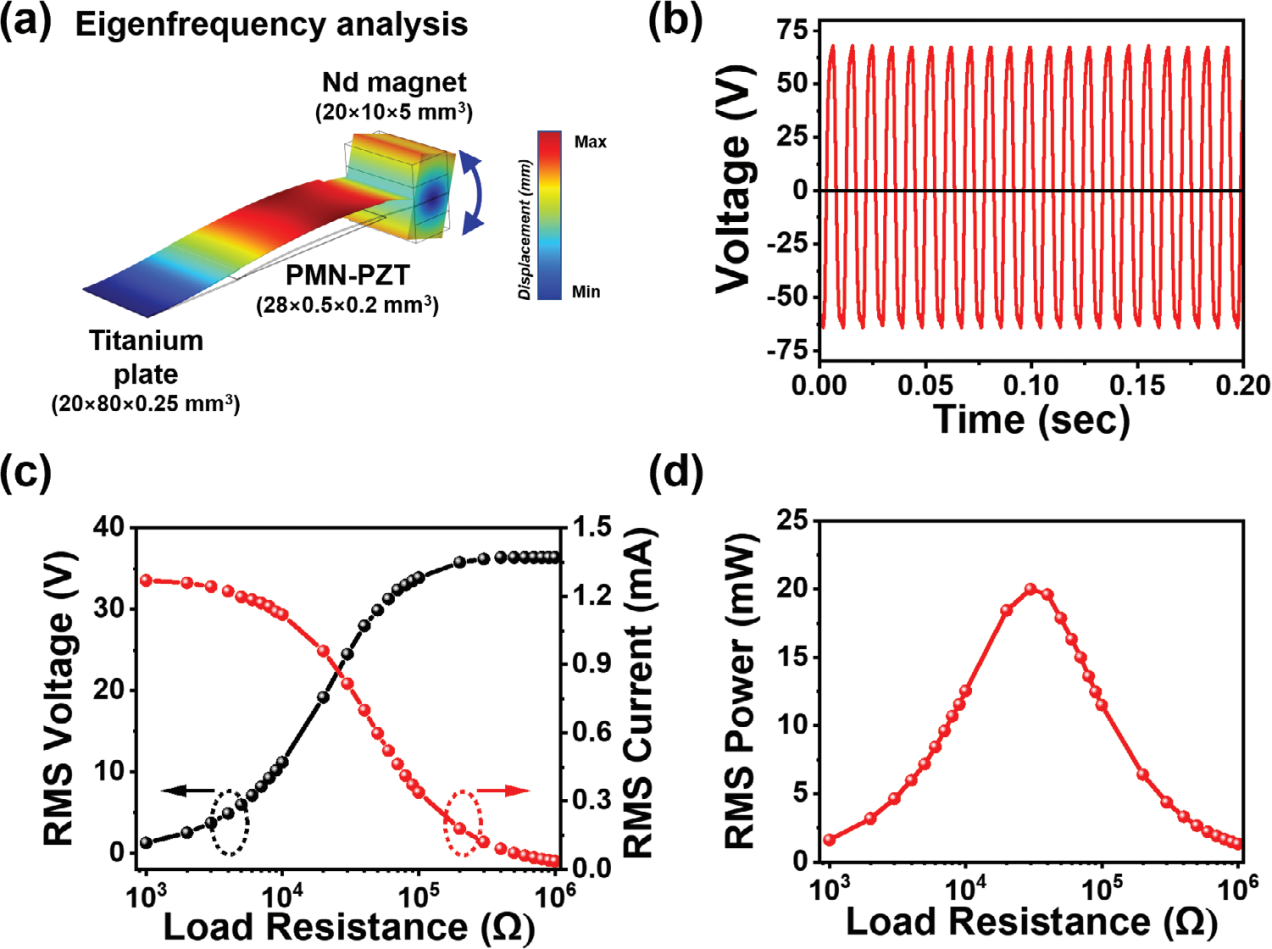
a) Second harmonic bending deformation of the cantilever‐structured piezoelectric MME generator analyzed by FEA. The cantilever beam shows the largest bending amplitude in the central part, and the proof magnet mass makes the pivotal swing motion. b) Time‐dependent open circuit voltage from the piezoelectric layer in the MME generator of 132 V_pp_. c) Root mean square (RMS) voltage and RMS current, and d) calculated power as a function of load resistance from 1 kΩ to 1 MΩ. The maximum RMS power of 20 mW was obtained at 30 kΩ.

In the aforementioned structure, the resonance bending vibration of the cantilever beam represents a fundamental motion in hybrid MME harvesting. Both the motion of the second harmonic resonance, which yields the largest displacement of vibration at the mid‐region of the cantilever structure, and the eigenfrequency of the MME cantilever were identified through FEA, as shown in **Figures** **2**a and S1 (Supporting Information). Figure S1a and Video S1 (Supporting Information) show the FEA results on the vibration mode changes of the cantilever beam with varying driving frequencies, particularly highlighting the first and second harmonic bending vibration modes, respectively. In the context of typical cantilever beams used in various applications, such as piezoelectric bending actuators, the vibration mode of the cantilever beam is strongly dependent on the excitation frequency. In particular, the second harmonic bending vibration mode exhibited maximal vibration displacement along the thickness direction, concentrated at the center of the beam. Conversely, in the case of the first harmonic bending vibration mode, maximal vibration displacement occurred at the end of the cantilever structure. The motion of the second harmonic bending mode manifested as concave and convex shapes of deflection at the center of the beam, wherein the proof magnet mass attached to the beam underwent pivotal swinging rather than linear motion along the thickness direction. These characteristics are advantageous for facilitating the hybridization of the EM induction coil and TENG with the bending beam. When the EM induction coil was coupled with the second harmonic bending mode of the MME generator, the proof magnet mass located at the end of the cantilever beam exhibited minimal displacement, thus necessitating a small volume for the magnet near the EM induction coil. Moreover, integrating the MME device with a TENG would be advantageous, leveraging the substantial deflection at the center of the beam without severe modification of the device structure.

Figure S1b (Supporting Information) shows the photographs and FEA results corresponding to various attachment positions of the piezoelectric SFC on the Ni cantilever plate for the vibrating cantilever structure in the piezoelectric MME generator to investigate the relationship between the positioning of the piezoelectric SFC and resulting piezoelectric output power. Figure S1c,d (Supporting Information) shows the results of the output voltages and root mean square (RMS) powers obtained from the fabricated piezoelectric MME generator, wherein an elastic Ni substrate was utilized, in relation to the positioning of the SFC under the first and second harmonic bending modes, respectively. One end of the cantilever was clamped with the non‐magnetic insulating rig blocks. A uniform AC magnetic field of 10 Oe was applied at each frequency for the first and second harmonic bending modes, which was achieved utilizing a Helmholtz coil driven by a function generator (WF1946a, NF corporation, Japan) and a high‐speed bipolar amplifier (HAS 4025, NF corporation, Japan).

In the case of the first harmonic bending mode (Figure S1c, Supporting Information), the highest output voltage and power were observed when the piezoelectric SFC was affixed at position 1, situated near the clamping end of the cantilever. Conversely, the output gradually decreased when the allocation position of the piezoelectric SFC was shifted to the opposite end of the beam. The maximum stress occurred at the clamping position during the first harmonic bending motion of the cantilever as well known; thus, the optimal power was obtained when the piezoelectric SFC was located at position 1.^[^ ^42^, ^43^, ^44^ ^]^ In the case of the piezoelectric MME generator operating under the second harmonic bending mode, optimal voltage, and power output were achieved when the SFC was attached at position 2, as shown in Figure S1d (Supporting Information). The maximum RMS power from the piezoelectric MME under the second harmonic bending mode was ≈30 mW, which was more than 12‐fold larger than that under the first harmonic bending mode (2.3 mW). Another advantage of the second harmonic bending mode compared with the first mode is the reduced matched load resistance. One of the primary challenges encountered in piezoelectric or triboelectric‐based energy harvesting is the impedance mismatch with the power management circuit or electrical load. Although the matched load resistance remained higher compared with that of the EM induction part, the second harmonic bending mode of MME generation significantly reduced this issue compared with the first harmonic bending mode. This reduction in matched load resistance can simplify the power management circuit. Therefore, for optimal piezoelectric effect, attaching a piezoelectric layer to the center of the cantilever beam in the MME generator is most advantageous. Previous studies have commonly employed magnetostrictive nickel (Ni) plates to exploit the magnetoelectric effect in the MME generator. However, although Ni plates demonstrate superior harvesting performance compared with other non‐magnetic metal plates, the mechanical fatigue properties of Ni are insufficient to ensure long‐term stability.^[^ ^44^ ^]^ Consequently, elastic Ti metal plates after minor design tailoring were chosen for further investigation in our study.

After optimizing the position of SFC in the MME cantilever beam, the central width of the Ti metal cantilever beam was configured as concave to increase the amplitude of central deflection under the second harmonic bending mode. A comprehensive summary of the piezoelectric MME harvesting performance is shown in Figure 2b–d. The measurement conditions were the same as previously described, with the only variation being the driving frequency.

Following the adjustment of the Ti‐based MME generator to operate under the actual second harmonic bending condition at 87 Hz, the maximum voltage output from the piezoelectric MME generator was obtained, as shown in Figure 2b. The maximum open circuit voltage was 132 V_pp_, with a pristine sinusoidal waveform. To characterize the RMS output power of the MME generator, the RMS voltage (*V_rms_*) was measured across various external load resistances, ranging from 1 kΩ to 1 MΩ at an AC magnetic field of 10 Oe, as shown in Figure 2c. The RMS output power (*P_rm_*_s_) of the MME generator was calculated by the following equation: *P_rms_ = V_rms_^2^/R* (where *R* is the external load resistance). A maximum *P_rms_* value of 20 mW was obtained at a load resistance of 30 kΩ, as shown in Figure 2d. The power observed in the Ti‐based MME generator was slightly reduced compared with that in its Ni‐based counterpart and can be attributed to the absence of the magnetoelectric effect in the MME cantilever beam.

### 2.2 Hybridization of EM Induction and Triboelectric Effect

To enhance the output power of the optimized hybrid MME generator, an additional Cu solenoid coil was introduced to depict the EM induction part of the hybrid MME generator. For EM induction harvesting, relative motion between the proof magnet mass and Cu coil could modify the magnetic field distribution in the EM coil. The electromotive force (*e*) derived by the electromagnetic induction effect is expressed by Faraday's law of induction, as follows.

(1) $$e=-\frac{d\Phi }{dt}$$

*Φ* is the magnetic flux perpendicular to the metal solenoid and *t* is the time. The induced *e* in a closed loop is equal to the alternation of the magnetic flux in unit time through the loop. As *Φ* is expressed by the equation of *B·A* (where *B* and *A* are the magnetic flux density and area vector, respectively), the *Φ* value is proportional to the *B* value.

**Figure 3:**
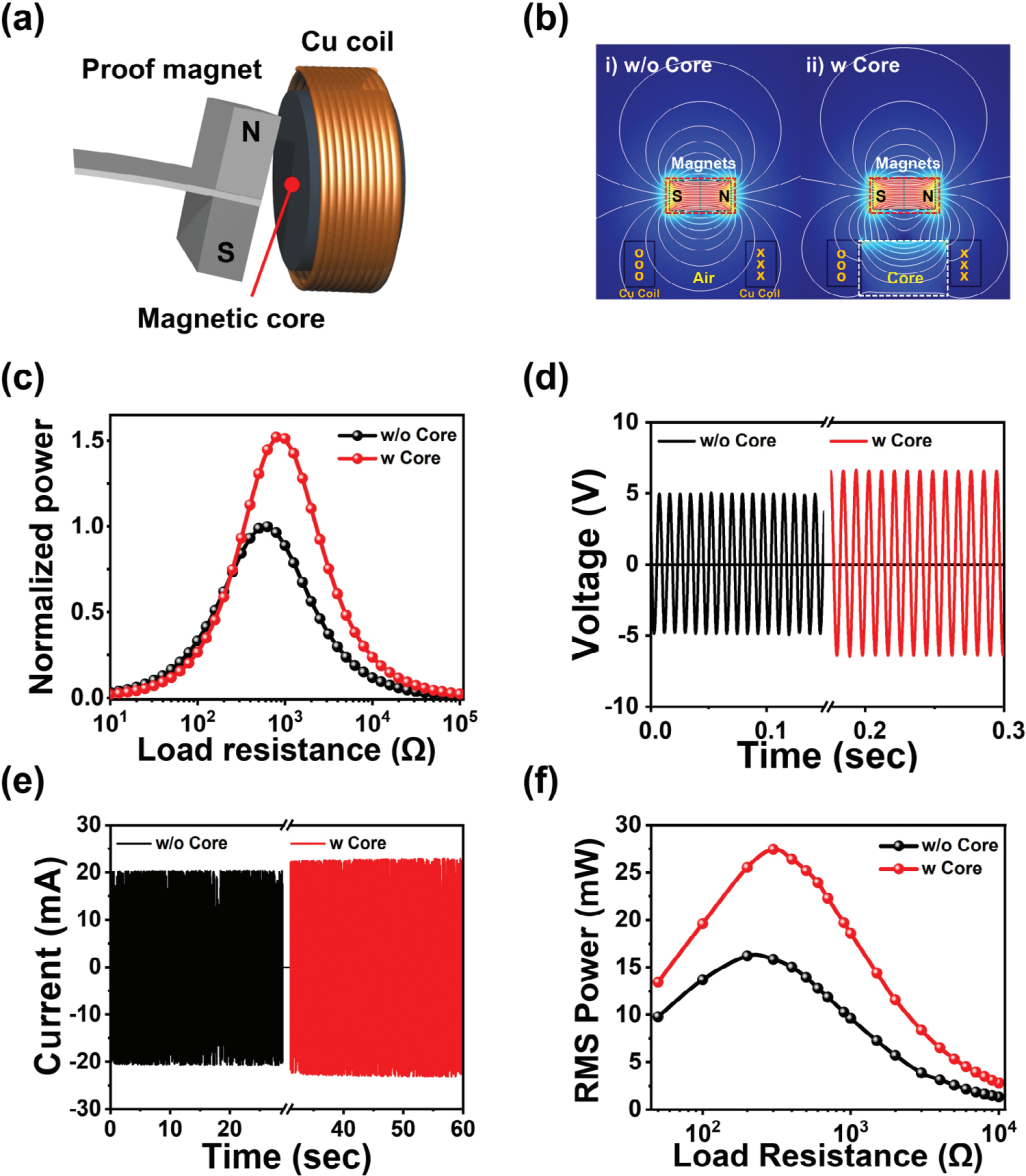
a) A schematic representation of the proof magnet mass at the end of the piezoelectric MME generator under the second harmonic bending mode, Cu solenoid EM coil, and magnetic core inserted into the hole of the EM coil. b) The FEA result of magnetic flux variations between the electromagnetic coil and magnets with/without a magnetic core for magnetic flux concentration. c) Normalized output power from the EM coil with and without the magnetic core in the FEA. d,e) Time‐dependent open circuit voltage and short circuit current signals of the Cu coil with and without the magnetic core. f) Calculated RMS output power values from the EM coil with and without the magnetic core as a function of the load resistance ranging from 50 Ω to 10 kΩ.

From Equation (1), the *e* value can be enhanced by a higher *B* value near the coil during the EM induction process. In this context, the incorporation of the magnetic flux concentrator, referred to as the magnetic core, into the hole area of the Cu solenoid EM coil could lead to the concentration of the magnetic field induced by pivotal swinging magnets near the EM coil, thereby improving the electric output of the EM induction part of the hybrid energy harvester. **Figure** **3**a shows the schematic illustration of the magnetic core positioned inside the Cu coil to enhance EM induction harvesting. In our design of the MME generator, the direction of the poles of the magnets is perpendicular to the axis of the Cu solenoid coil. This perpendicular orientation of the magnet's poles relative to the coil axis is intentionally chosen to optimize the EM induction process within our specific setup (pivotal motion of the magnet in second harmonic vibration of MME generator). Notably, the effectiveness of the magnetic flux concentration is heavily influenced by the permeability of the magnetic core materials. A cylindrical high‐permeability magnetic core, constructed from steel structure 400 (SS 400, relative permeability: 1300), is located inside the Cu solenoid to amplify the induced magnetic flux density on the coil.^[^ ^45^ ^]^ The comparison of the normalized magnetic flux density distributions with and without the magnetic concentrator at the core area of the Cu coil is depicted in the FEA results for the EM induction part in Figure 3b. The repeated clockwise and anticlockwise pivotal vibration motions of proof mass magnets were replicated to emulate the movement on the cantilever structure at the second harmonic resonance bending mode. As the magnetic flux density value on the coil was significantly lower than that near the magnet area, the normalized magnetic flux density distributions in the coils are individually shown in Figure S2 (Supporting Information). The sample with the magnetic core exhibited higher normalized magnetic flux density values not only in the coil but also at the center area of the coil compared with that without the magnetic core.

Figure 3c shows the normalized output power estimated by FEA for the sample with and without the magnetic core on the EM induction part. In the FEA simulation, the normalized output power of the structure with the core was 150% higher compared with that of the configuration without the core. The solenoid had a conductivity of 5.8 × 10^7^ S/m and a relative permeability of 0.999994, and the magnets showed a remnant flux density of 0.35 T and recoil permeability of 1.05 in the theoretical FEA modeling. As the magnetic flux density *B* is directly proportional to the magnetic permeability of the core material inside the Cu solenoid, the change rate of magnetic flux (*dΦ/dt*) of the magnetic core inside the EM coil was higher compared with that in the air (has relative permeability of ≈1), thus enhancing the maximum output power at an optimum resistance in FEA.

Additionally, the core volume also plays a significant role in influencing the flux density of the coil. Figure S3 (Supporting Information) shows the normalized output power obtained by FEA modeling and experimental output properties with and without the presence of a magnetic flux‐concentrating core. To analyze the effect of core volume, the thickness of the magnetic core was changed upon insertion into the EM induction coil. We found that the EM induction behavior exhibited gradual enhancement with increasing thickness of the magnetic core, in contrast to the performance of the Cu coil without the magnetic flux concentrator. When the magnetic core exceeded the critical thickness, the attraction force between the tip of the magnet of the MME generator and the magnetic core restrained the oscillation of cantilever; therefore, a thickness of 18 mm, which is nearly the same as the length of the Cu solenoid coil, was determined to be optimal for the EM induction part. Through the preliminary investigation, we established the optimized diameter and thickness, as well as the optimal core material type. Subsequent measurements were conducted under these specified conditions to evaluate the resulting outcomes.

Figure 3d–f shows the experimental harvesting results from the EM induction part to confirm the effect of the magnetic core in the Cu coil under the second harmonic resonance bending mode of the piezoelectric MME cantilever structure. The open circuit voltage and maximum short circuit current value of the EM induction part with the magnetic flux concentration core were 13 V_pp_ and 23.0 mA, respectively, whereas those of the sample without the magnetic flux concentration core were 9.8 V_pp_ and 20.6 mA, respectively. Under the aforementioned conditions of the maximum output, the RMS powers were measured in a range from 50 Ω to 10 kΩ. The maximum *P_rms_* value of the magnetic flux concentration core‐installed EM induction part within the hybrid MME generator was 27.5 mW at an optimum load resistance of 300 Ω, which was 170% higher compared with that of the counterpart without the core (maximum *P_rms_* value of 16.3 mW at 300 Ω). This result indicates that the magnetic core for the flux concentration in the Cu solenoid noticeably contributes to enhancing the electric power of electromagnetically induced energy during the MME operation.

The energy conversion efficiency of an EM induction generator is influenced by several factors. While the number of turns in the coil contributes to the induced electromotive force (EMF), it is not the sole factor impacting efficiency. The efficiency of EM generators is also significantly affected by the implementation of speed‐increasing mechanisms, such as resonance frequency operation, which can substantially boost overall performance despite the potential for increased mechanical damping. By tuning the MME system to operate at its resonance frequency, the relative motion between the magnets and the coil is maximized through the enhanced displacement of the up‐and‐down vibrations of the cantilever structure, leading to a significant increase in output power. To underscore the positive effects of resonance frequency operation in the hybrid MME generator, we compared the output performance of the MME generators in both resonance and off‐resonance modes, demonstrating significantly higher output signals in the resonance mode as present in Figure S4 (Supporting Information).

In the pursuit of maximizing the output power, the integration of TENG within the hybrid MME generator presents a promising avenue. TENG exhibits structural versatility, enabling its seamless integration with the piezoelectric MME generator, and demonstrates efficient electricity generation from mechanical energy sources.^[^ ^5^, ^7^, ^15^, ^18^, ^19^, ^46^, ^47^, ^48^, ^49^ ^]^

**Figure 4:**
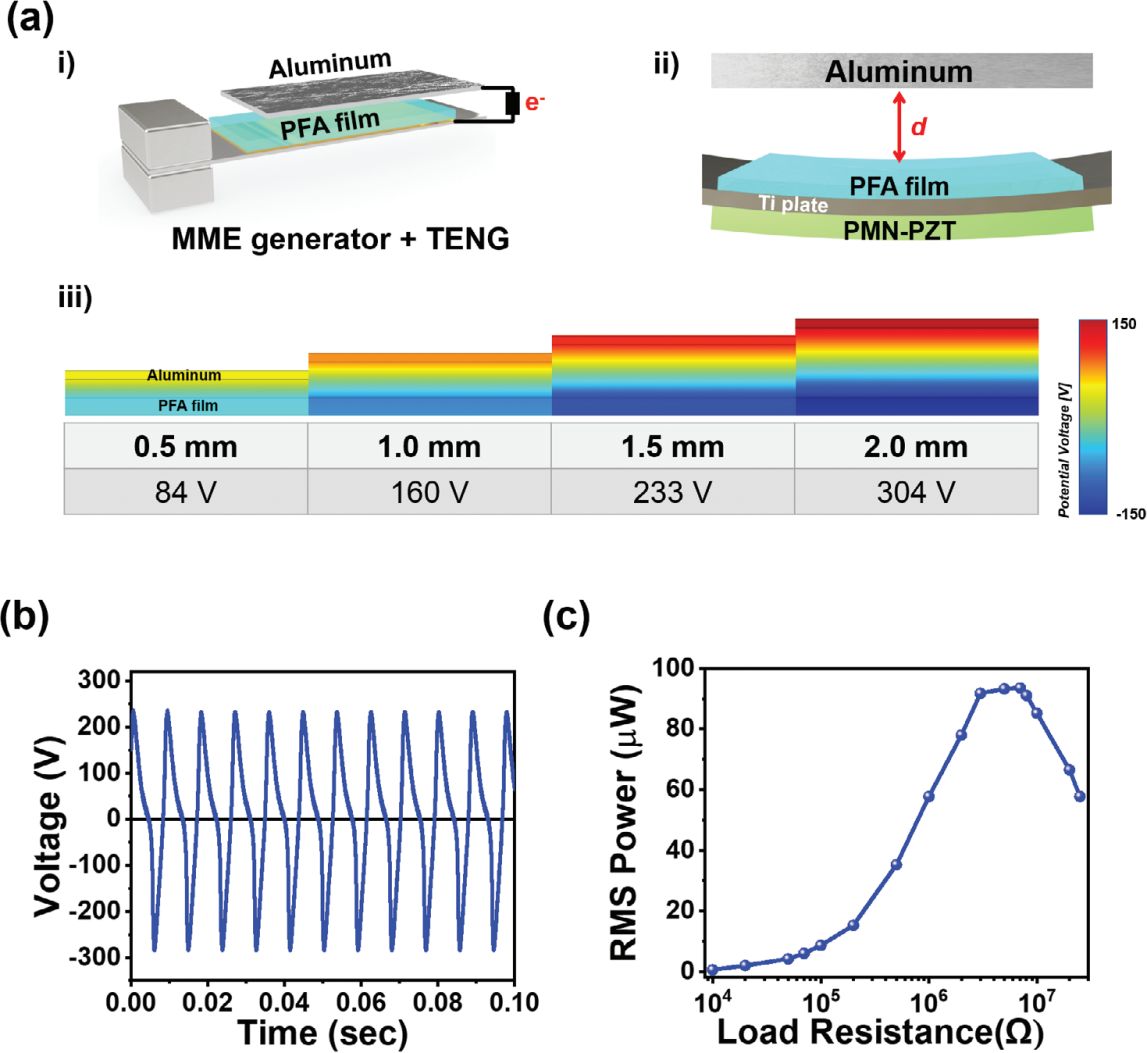
a) i) Schematic of the integration of the MME generator and TENG comprising an aluminum foil and a platinum‐electrode‐treated PFA film; ii) Illustration of the distance between the aluminum foil and PFA film in a TENG with the contact mode for FEA; iii) The increase in the electric potential difference with distance from 0.5 to 2.0 mm between the aluminum foil and PFA film. b) Time‐dependent open circuit voltage of the triboelectric generator, with a maximum of 520 V_pp_ c) Calculated power of the TENG with respect to the load resistance ranging from 10 kΩ to 10 MΩ (94 µW at a load resistance of 7 MΩ).

In the phenomenon of triboelectric effect, a material acquires an electric charge upon contact with another material through friction, facilitating interfacial charge transfer. Therefore, selecting two materials with a significant difference in charge density is advantageous.^[^ ^39^, ^50^ ^]^ In our study, we selected a PFA film and Al foil from the reported triboelectric series and integrated them into the hybrid MME harvesting structure. These materials are easy to acquire and can be conveniently manufactured in thin configurations, enabling a simple attachment process and position adjustment. Additionally, in our hybridization process, the PFA film was affixed to the opposite side of the cantilever beam with the piezoelectric layer. This approach ensures that the bending is uniform and concurrently occurs during the second harmonic resonance without canceling the vibration in the MME generator, as illustrated in **Figure** **4**a.

The schematic in Figure 4a–i shows the integration of the dielectric (PFA)‐metal (Al) TENG into the second harmonic bending‐based piezoelectric MME generator operating in the contact‐separation mode under the influence of an external magnetic field. As the two surfaces come into contact and subsequently separate, a small gap (*d*) is formed, thus leading to the generation of a potential drop. When the PFA film and Al foil come into contact with the oscillation of the MME beam, free electrons from one electrode migrate to the other electrode to establish an opposite potential and counterbalance the electrostatic field. Once the gap is closed, the potential created by the triboelectric charge dissipates and the electrons flow back.^[^ ^7^, ^39^, ^50^, ^51^ ^]^

The separation distance (*d*) between the two materials serves as a variable parameter influencing charge generation. The theoretical potential was modeled by FEA using properties, such as the thickness, distance change, and surface charge density of the two materials, which were then validated through experimental measurement following the hybridization design. As illustrated in Figure 4a–ii, the vibration of the cantilever structure, including the PFA film, results in a fluctuation in the distance between the PFA film and Al foil. The FEA result shown in Figure 4a–iii indicates the escalation in the electrostatic potential value with an increase in the distance (*d)* between the two layers. Under the second harmonic bending mode, when the two triboelectric layers begin to fall and the gap increases, the distance between the concave and convex motions can be arbitrarily determined to confirm the triboelectric potential by dividing it into four stages. As the *d* varied from 0.5 to 2.0 mm, the potential difference between the Al foil and PFA film increased to 84, 160, 233, and 304 V, respectively, with an increasing gap distance.

Notably, the open circuit voltage from TENG can be obtained by the following equation.

(2) $$V=\sigma \frac{d}{\varepsilon_{0}}$$

*ε_0_* is the permittivity of free space, *d* is the separated distance between the PFA film and Al foil, and *σ* is the triboelectric charge density. To optimize the performance of the triboelectric component in the hybrid MME generator, we investigated the effect of varying the gap distance between the PFA film and Al foil. The gap distance was systematically adjusted from 1 to 4 mm while monitoring the corresponding open‐circuit output voltage signals. As shown in Figure S5 (Supporting Information), a gap distance of 2 mm provided the highest output performance from the TENG. This optimized gap distance was found to maximize the efficiency of the contact‐separation mode operation. Figure 4b,c shows the measured output voltage and power from the TENG hybridized with the MME generator. The continuous semi‐triangular waveform of 520 V_pp_ was obtained owing to triboelectrification. In contrast to other TENGs that can generate intermittent spike‐type voltages, this continuous semi‐triangular waveform persists as it is constantly charged and discharged under the second harmonic resonance condition. Consequently, the results of the output energy calculated by power integration over time are more favorable compared with those of other TENG devices.

The *P_rms_* value was measured as a function of the external load resistance in the range from 10 kΩ to 25 MΩ. A maximum *P_rms_* value of 94 µW was extracted at a load resistance of 7 MΩ. Despite the ability of TENG to serve as an additional power source without significant structural modification, the power output of TENG was considerably low under high matched impedance compared with those of MME and EM harvesting.

To evaluate the long‐term stability and reliability of the hybrid MME generator, we conducted durability tests involving 10^7^ cycles of up and down vibration motions of the cantilever structure as shown in Figure S6 (Supporting Information). The results showed that the piezoelectric, EM induction, and triboelectric components maintained stable open‐circuit output voltage signals throughout the testing period, with no observed degradation in performance or damage to the device. These findings confirm the hybrid MME generator's robustness and suitability for extended use in energy harvesting applications.

### 2.3 Driving of Wi‐Fi IoT Sensor

**Figure 5:**
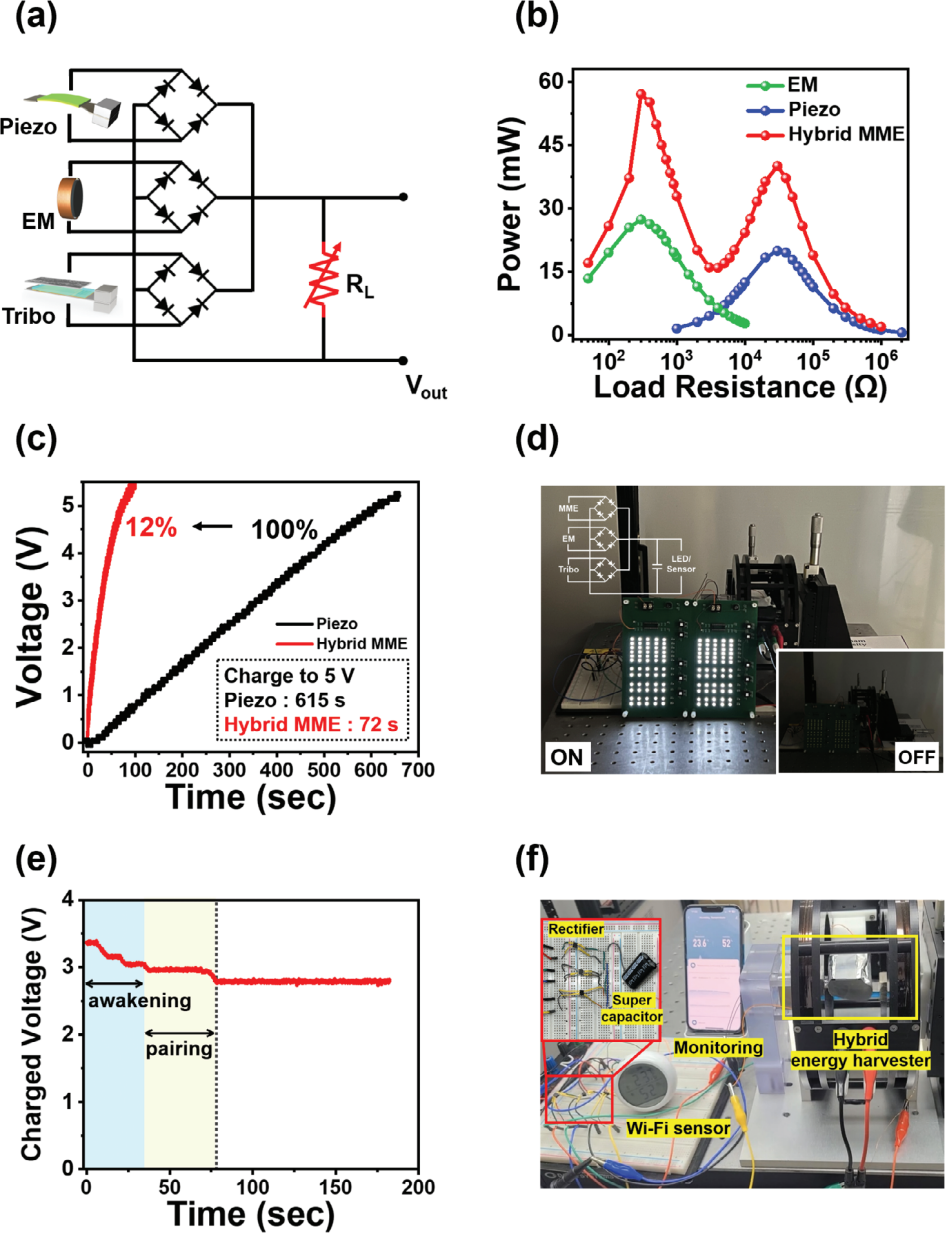
a) Circuit schematic for the characterization of the integrated hybrid energy harvester. The output powers from each energy harvester were independently rectified based on AC‐DC by three full‐bridge rectifiers and connected to the load resistance in parallel. b) The RMS power and rectified parallel sum power are obtained by combining each rectified output power with changing load resistances. The appearances of impedance matching occurred twice, namely, at 300 Ω (57 mW) and 30 kΩ (40 mW). c) Time‐dependent 0.1 F supercapacitor charging behavior of the sole MME generator and hybrid configuration. The hybrid energy harvester could reduce the time by 88% compared with that required to charge the capacitor using the MME generator. d) Photograph of the 120 light‐emitting diodes (LEDs) powered by the hybrid energy generator. e) Voltage graph of the 6.0 F supercapacitor driving a Wi‐Fi sensor powered by the hybrid energy harvester. f) Photograph of the multifunctional Wi‐Fi sensor being driven by the hybrid energy harvester. The moment the Wi‐Fi sensor communicates with the mobile after charging the 6.0 F supercapacitor with the hybrid energy harvester. Videos are provided in Supporting Information.

To enable the operation of the Wi‐Fi IoT sensor by hybridizing the three energy harvesting mechanisms, we evaluated the integrated output power by rectifying the output powers from each harvester. Output powers of ≈20 mW, 27.5 mW, and 94 µW were extracted from the piezoelectric MME, EM induction, and TENG parts and these have ratios of 42%, 57.8%, and 0.2%, respectively. As the output powers from each harvester may have different phases, they were individually rectified based on AC‐DC and connected in parallel, and the integrated DC power was then measured across a range of external load resistances from 50 Ω to 1 MΩ, as shown in **Figure** **5**a. Interestingly, two optimal load resistances were identified for maximum power extraction. One optimal load resistance corresponded to a power of 57 mW at the matching impedance value (300 Ω) of the EM harvester, whereas the other corresponded to a power of 40 mW at the matching impedance value (30 kΩ) of the piezoelectric MME harvester, as shown in Figure 5b. Notably, these values exceeded the simple arithmetic sum of the three RMS power values. This synergetic effect of hybridization might be related to the phase differences of each output power from the harvesters. Although the output powers from each harvesters were rectified, the voltage and current are not completely flattened since there are no regulating capacitors as shown in Figure S7 (Supporting Information). It is believed that slight difference of voltage and current phases from piezoelectric MME generator and EM coil can make this higher output power from the hybrid generator. When the voltages are connected in parallel after each rectification of piezoelecectic MME and EM harvester, the zero point of the half‐sine graph disappears and the minimum voltage point also rises to more than 10 V, which is referred to in Figure S7a (Supporting Information). In addition, the rectified sum voltages at load resistances of 300 Ω and 30 kΩ, which are points where impedance matching of EM and MME generator are compared with their respective voltages and are shown in Figure S7b,c (Supporting Information). The rectified sum voltage connected in parallel has a larger voltage than the single harvester, and the power greatly amplified at the two impedance matching points is advantageous for autonomous power supply for IoT sensors.

Figure 5c shows the time‐dependent charging behavior of 0.1 F supercapacitor to 5 V by the hybrid energy harvester. We observed that the hybrid energy harvester achieved a charging time of 72 s, representing a reduction in time of ≈88% compared with that of the piezoelectric MME single‐harvesting system (615 s). We confirmed that the three signals past the full bridge rectifiers can be combined to directly turn on the 120 high‐intensity light‐emitting diodes (LEDs), as shown in Figure 5d and Video S2 (Supporting Information). Furthermore, the Wi‐Fi multifunctional sensor (TS‐H7, Unicorn IoT) could be powered by charging the 6.0 F capacitor connected to the rectifying circuit, as shown in Figure 5e,f. Upon direct connection to the 6.0 F supercapacitor and charging up to 3.4 V, the sensor initializes its running. The operation of the Wi‐Fi IoT sensors consumed a substantial amount of power at once, and the voltage stored in the supercapacitor decreased to 3.0 V. Subsequently, during the process of searching and pairing with a mobile device, the voltage further decreased to 2.8 V. However, once the connection was established, the Wi‐Fi sensor operated in the low‐power communication mode, and the hybrid energy harvester provided an environment for stable communication, while also maintaining the power supplied to the supercapacitor. The Wi‐Fi multifunctional sensor (temperature and humidity) required up to 300 mW of instantaneous maximum power. This was confirmed by connecting the sensor to a source meter (2450, Keithley) for power calculation, as shown in Figure S8 (Supporting information). When it was initially activated, the sensor consumed a maximum peak power of up to 335 mW for device start‐up, even if it was instantaneous. During the process of pairing with a mobile device through Wi‐Fi communication, the sensor required a power of ≈310 mW. Subsequently, data were transmitted continuously every 10 s, with a monitored peak power consumption of ≈3 mW, and the power demand significantly decreased compared with that during the awakening and pairing stage. Video S3 (Supporting Information) shows the continuous driving of the Wi‐Fi multifunctional sensor by the MME‐EM‐TENG hybrid energy harvester, and the oscilloscope simultaneously monitors the voltage level of the capacitor.

These practical application cases demonstrate that hybrid energy harvesting can effectively provide sufficient power to supplement conventional MME generators, thus expanding the application field from Bluetooth to Wi‐Fi communication and beyond.

To demonstrate the expandability of the hybrid MME generator for other IoT applications, we conducted an additional practical test where the hybrid MME generator powered an IoT temperature sensor (EZ430‐RF2500, Texas Instruments) which uses low‐power radio‐frequency (RF) protocol for networking as shown in Figure S9 (Supporting Information). After charging a 0.1 mF supercapacitor to 3.0 V, the IoT temperature sensor successfully began operation and continuously transmitted temperature data to a monitoring system. This demonstration highlights the potential of the hybrid generator to enable self‐sustaining, maintenance‐free operation in various IoT devices, which is essential for the development of more autonomous and efficient IoT networks.

## 3 Conclusion

We described the hybridization of energy conversion mechanisms to overcome the current power limitation of single energy harvesting technology and discussed the use of hybrid energy conversion mechanisms as a power source for high‐power‐consuming devices, such as Wi‐Fi communication sensors. After optimizing the conditions for the piezoelectric MME generator, EM induction coil, and TENG to maximize power extraction, each energy harvesting component was integrated, and the output power of the hybrid MME generator was characterized. Upon individual rectification, followed by the integration of the output power, the MME system extracted powers more than the simple arithmetic sum of the three RMS power values and successfully powered a Wi‐Fi‐based temperature and humidity multifunctional sensor that required over 300 mW of driving power. Notably, it exhibited a stable power supply even during the high‐power startup and pairing processes, ensuring continuous and reliable communication.

## 4 Experimental Section

### Fabrication of the Piezoelectric MME Generator

To fabricate an MME generator comprising the piezoelectric material and super‐elastic plate, the Pb(Mg_1/3_Nb_2/3_)O_3_‐Pb(Zr,Ti)O_3_ (PMN‐PZT) single crystal fiber composite (SFC, 28 (l)× 14 (w)× 0.20 (t) mm^3^) (CSH1, Ceracomp. Co. Ltd., Korea) and titanium (Ti) plate (80 (l)× 20 (w)× 0.25 (t) mm^3^) were laminated with an epoxy adhesion layer (DP‐460, 3M, USA). PMN‐PZT single crystals were grown by the solid‐state single crystal growth (SSCG) method, and the plate form of PMN‐PZT single crystals was machined into rectangular fiber forms with dimensions of 28 (l)× 0.5 (w)× 0.20 (t) mm^3^ by the dicing saw. The detailed fabrication process for the SFC was described in our previous studies.^[^ ^24^, ^25^, ^26^ ^]^ The neodymium iron boron (NdFeB) permanent magnets, serving as the proof mass for the second harmonic bending mode vibration, were attached at the free end tip of the cantilever beam, which was clamped at the opposite end. The weight and position of the proof magnet mass can determine the natural second bending resonance frequency of the MME cantilever beam.

### Hybridizing the EM Induction Coil

Considering the formation of the solenoid coil for the EM induction component, an enamel‐coated Cu wire with a diameter of 200 µm was wound around a cylindrical plastic frame with dimensions of 24 mm (diameter) × 17 mm (length), with ≈2200 turns. The magnetic flux concentrating core, with a diameter of 22 mm, was inserted into the center hole of the solenoid coil with varying thicknesses of 6, 12, and 18 mm.

The EM induction coil with or without the magnetic concentration core was positioned at a distance of 4 mm from the magnet mass at the tip of the MME generator, as shown in Figure 1. The clockwise and counterclockwise pivotal swing vibrations of proof magnets on the cantilever structure under an external AC magnetic field alter the amount of magnetic flux into the solenoid coil, thereby leading to the generation of electric current in the coil by the EM induction effect.

### TENG Integrated into MME Generator

To integrate the TENG with the hybrid MME and EM energy harvester, a perfluoroalkoxy alkane (PFA) film (50 µm‐thick, Alphaflon, Korea) and aluminum (Al) foil (15 µm‐thick) were combined to create a contact‐type TENG‐MME generator. A platinum (Pt) electrode was coated by direct current (DC) sputtering on the back side of the PFA film, which served as the electronegative material, and was attached to the Ti plate of the MME by double‐sided adhesive tape. An Al foil was positioned above the PFA film to act as the electropositive layer for TENG. By utilizing the reciprocating second harmonic bending motion of the MME generator, the immovable Al layer, and PFA film on the MME generator periodically make contact and separate with a frequency matching that of the applied magnetic field. This process induces tribo‐charges generated by contact electrification between the inner surfaces. The design parameters, such as the stand‐off distance between the Al layer and PFA film, were optimized by finite element analysis (FEA; COMSOL Multiphysics) to achieve optimal TENG performance.

When a uniform AC magnetic field was generated by the Helmholtz coil, the MME generator, with one end clamped, oscillates and generates electricity. The EM coil located at the vicinity of the vibrating proof magnet mass at the end of the MME generator also generates induced electricity owing to the fluctuating magnetic flux over time. By integrating TENG into a region with maximum vibration between the fixed cantilever and proof magnetic mass ends of the MME structure, additional electrical energy can be generated without significant modification of the EM‐MME hybrid generator.

## Conflict of Interest

The authors declare no conflict of interest.

## Supporting information



Supporting Information

Supporting Information

Supporting Information

Supporting Information

## Data Availability

The data that support the findings of this study are available from the corresponding author upon reasonable request.
