# Supplementary material for: Driving Wi‐Fi IoT Sensors by a Hybrid Magneto‐Mechano‐Electric Energy Generator Extracting a Power of over 50 mW
Source: Adv Sci (Weinh). 2024 Sep 30;11(44):2405526. doi: 10.1002/advs.202405526 (PMC11600218; doi:10.1002/advs.202405526)
Supplement: Supplementary file 1 — Supporting Information [file ADVS-11-2405526-s004.pdf]

ADVANCED SCIENCE
Open Access

Supporting Information

for *Adv. Sci.*, DOI 10.1002/advs.202405526

Driving Wi-Fi IoT Sensors by a Hybrid Magneto-Mechano-Electric Energy Generator Extracting a Power of over 50 mW

Seungah Lee, Chang Min Baek, Gang Hyeon Kim, Srinivas Pattipaka, Hyunseok Song, Jongmoon Jang, Geon-Tae Hwang and Jungho Ryu**

Supporting Information

Driving Wi-Fi IoT sensors by a hybrid magneto-mechano-electric energy generator extracting a power of over 50 mW

Seungah Lee, Chang Min Baek, Gang Hyeon Kim, Srinivas Pattipaka, Hyunseok Song, Jongmoon Jang, Geon-Tae Hwang and Jungho Ryu**

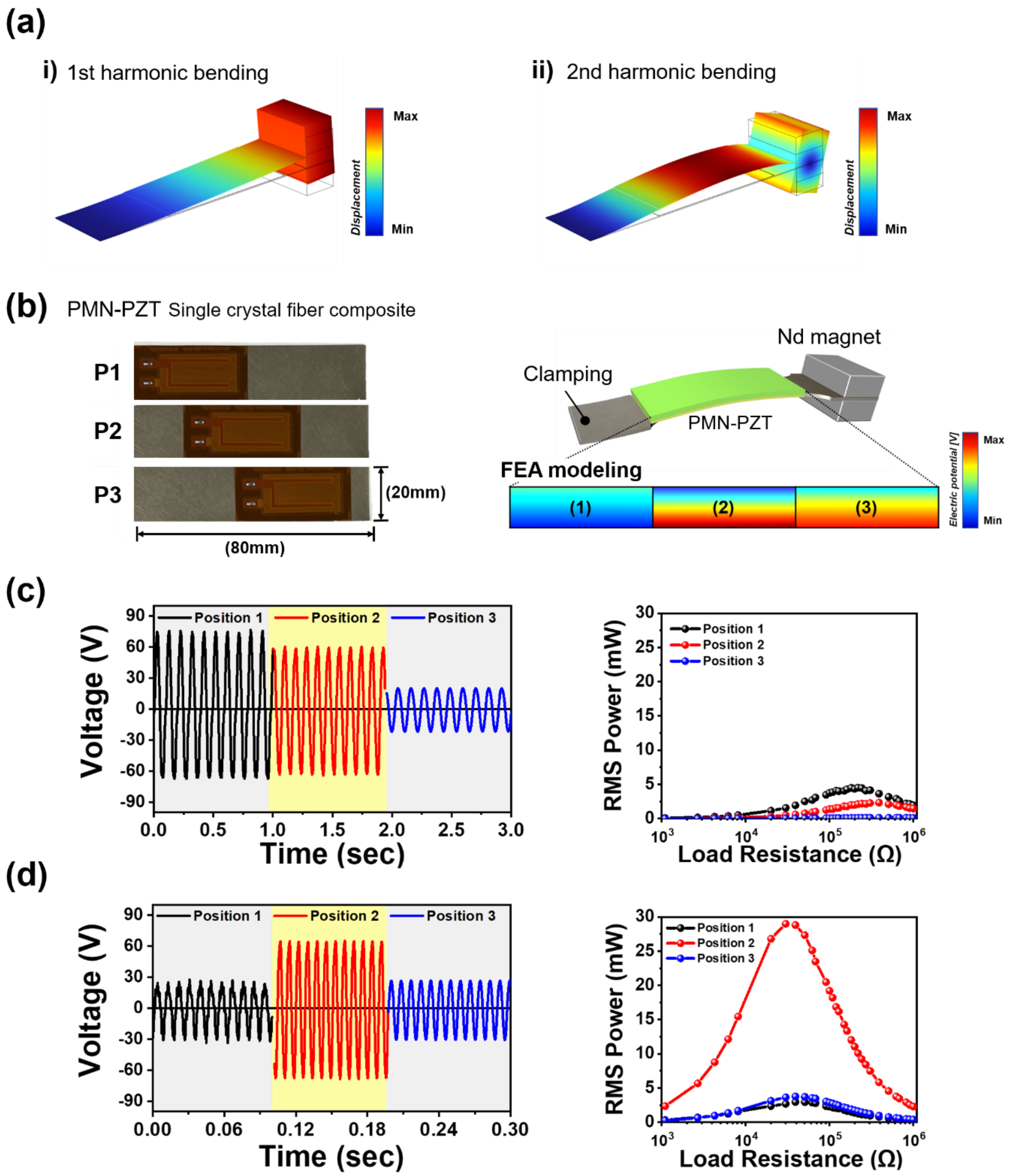

Figure S1. (a) i) 1st and ii) 2nd harmonic bending motion of piezoelectric MME generator from FEA modeling. (b) Photograph and schematic illustration of the piezoelectric single crystal fiber composite (SFC) on a Nickel plate divided into three different positions. Comparison of electric potential of single crystal in 2nd harmonic bending mode from FEA modeling. (c)-(d) Time-dependent voltage from 1st and 2nd harmonic bending motion of piezoelectric MME generators in open circuit condition according to position of piezoelectric SFC. Calculated RMS power of 1st and 2nd harmonic bending motion under the load resistance from 1 kΩ to 1 MΩ.

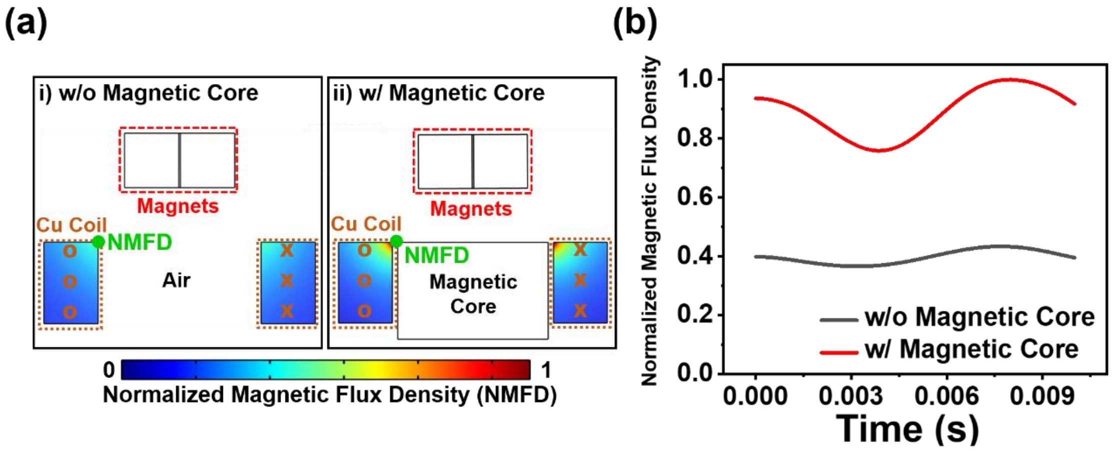

Figure S2. (a) FEA simulation model illustrating the normalized magnetic flux density (NMFD) distribution in the Cu coil area with and without a magnetic field concentrator inside the coil. (b) NMFD values in the edge point of Cu coil as a function of time with and without a magnetic flux concentrator.

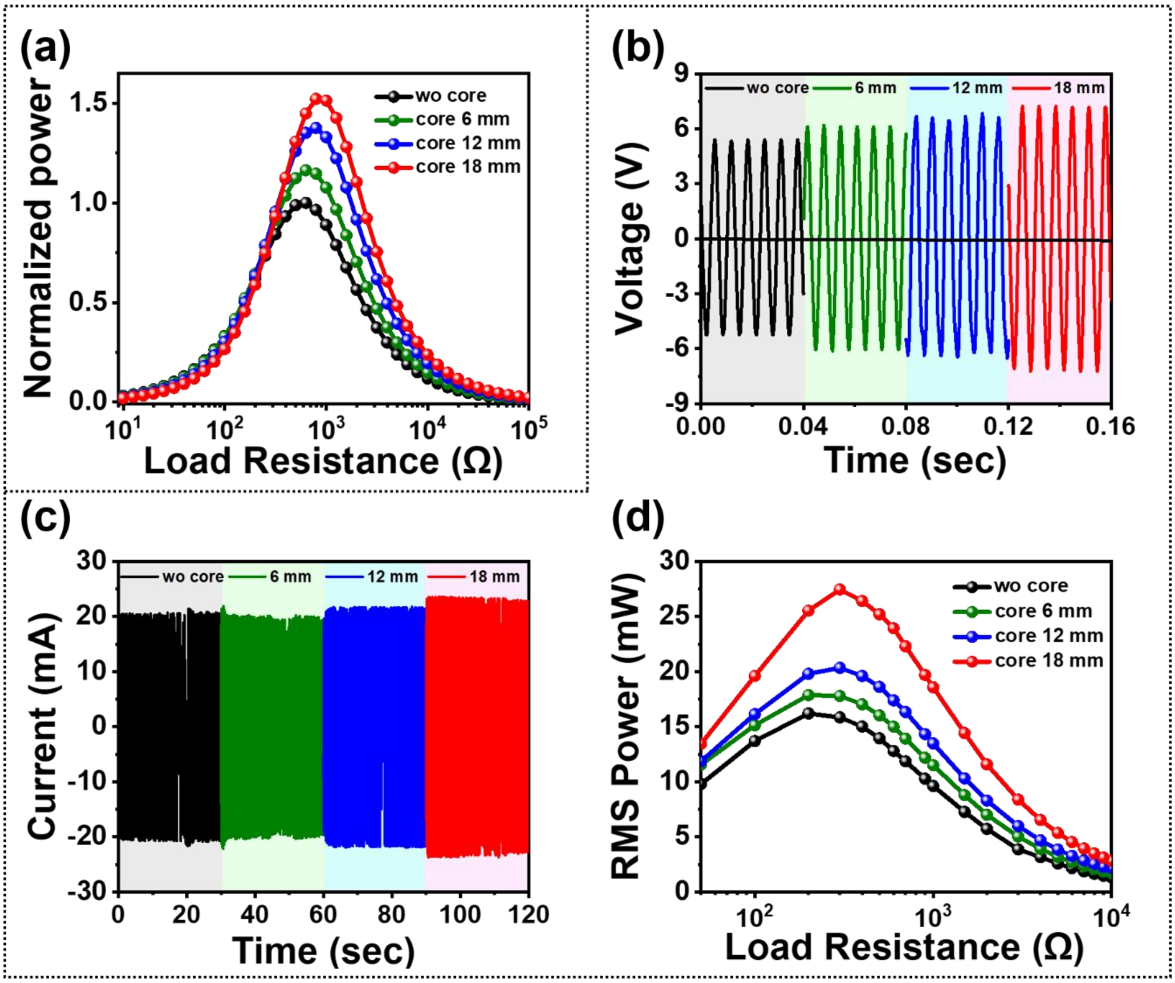

Figure S3. (a) The normalized output power obtained by FEA modeling and experimental output properties with and without the presence of a magnetic flux-concentrating core. (b)-(c) Time-dependent open circuit voltage, short circuit current of copper EM coil. (d) Calculated power of the coil under the load resistance from 50 Ω to 10 kΩ.

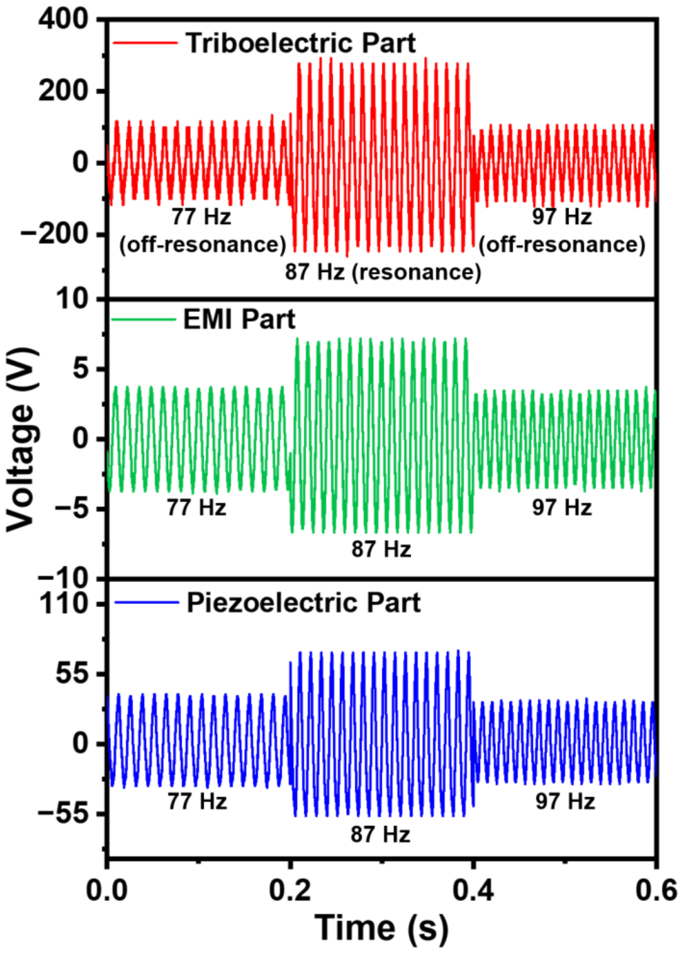

Figure S4. Measured open-circuit voltage signals from the piezoelectric, EM induction, and triboelectric components of the hybrid MME generator under resonance frequency (87 Hz) and off-resonance frequency conditions (77 Hz and 97 Hz).

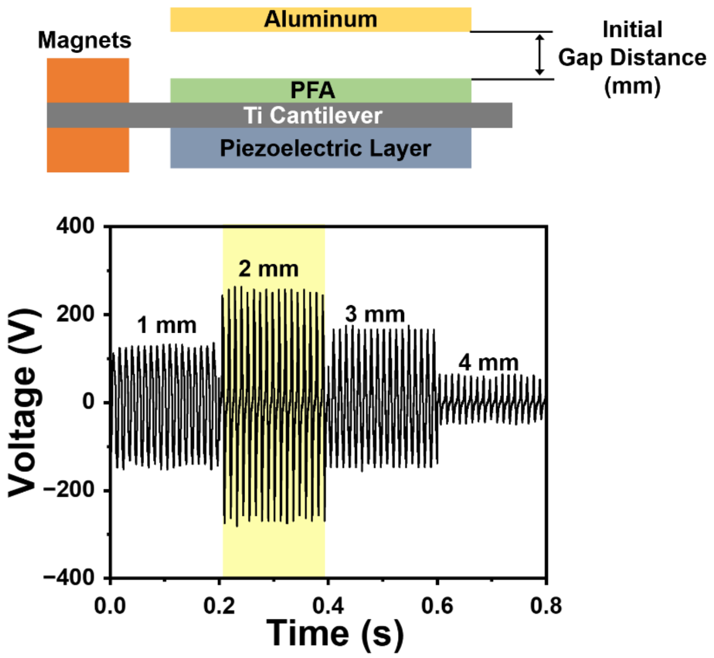

Figure S5. Open-circuit output voltage signals from the triboelectric component of the hybrid MME generator as a function of the initial gap distance between the PFA film and Al foil.

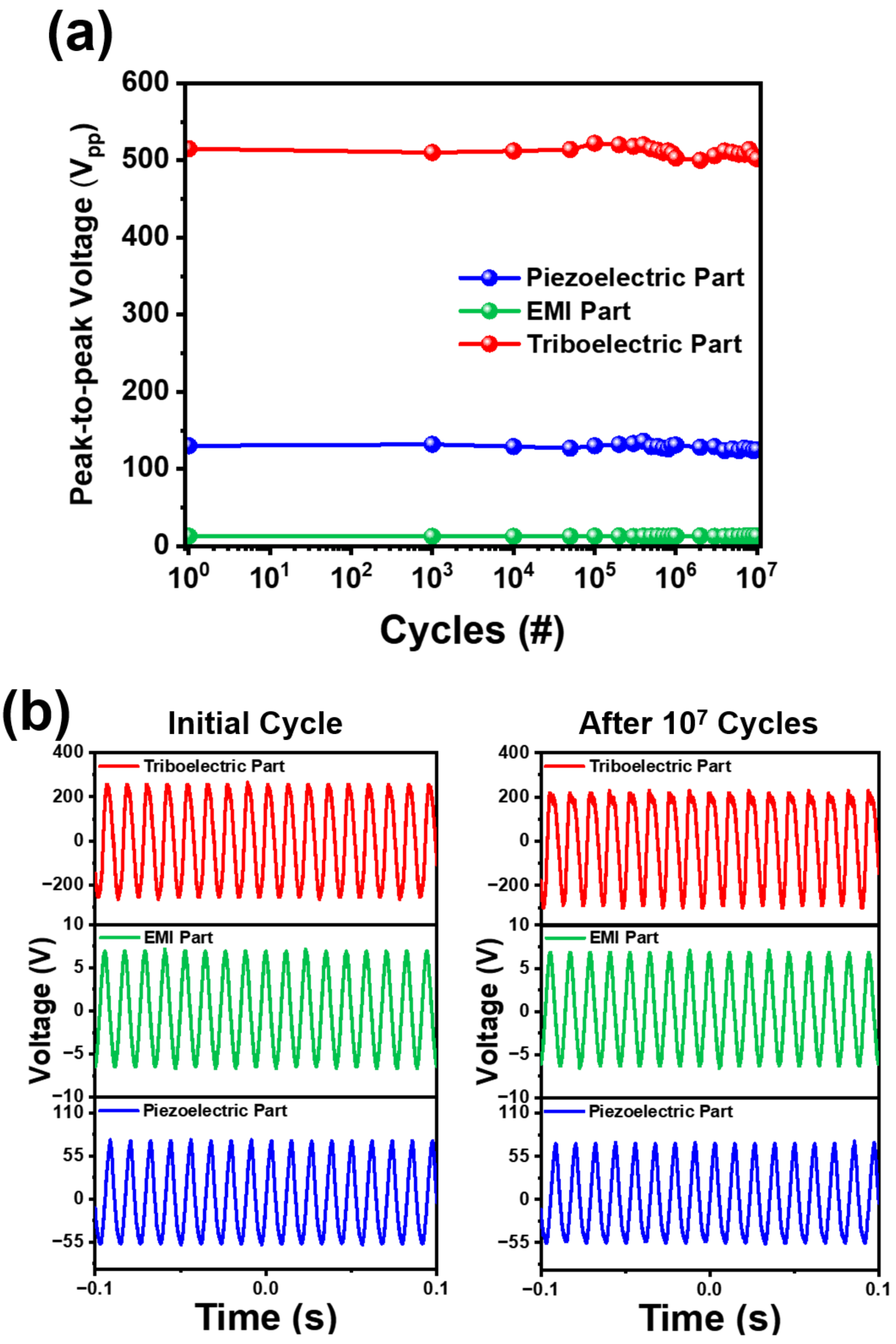

Figure S6. (a) Measured peak-to-peak open-circuit voltage signals from the hybrid MME generator during the long-term stability test, conducted over 10^7 cycles of vibration. (b) Comparison of output voltage signals from the piezoelectric, EM induction, and triboelectric components at initial operation and after 10^7 cycles, demonstrating stable performance and reliability across all parts.

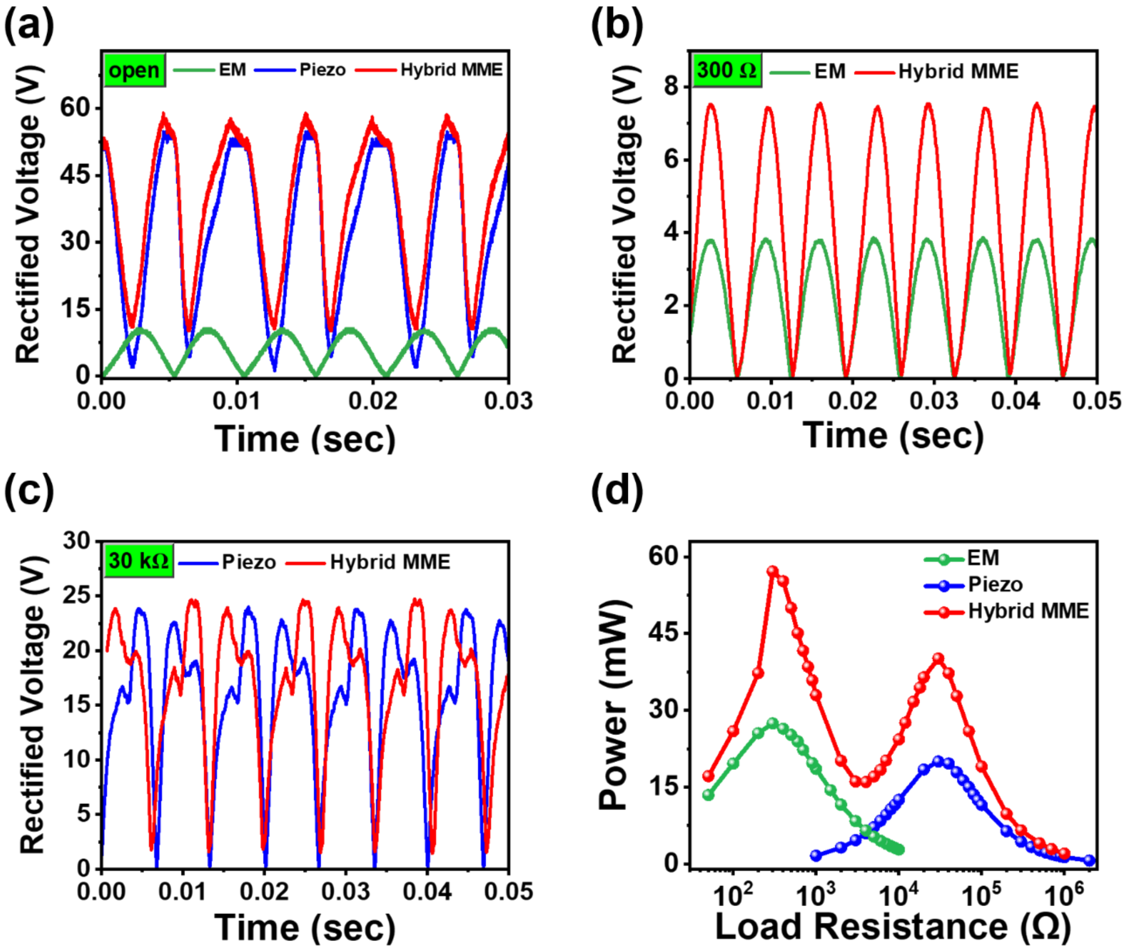

Figure S7. (a) Rectified open circuit voltage of the piezoelectric MME and EM energy harvester, as well as the sum of the two rectified voltages from the hybrid MME harvester. The rectified waveform voltages from the piezoelectric MME harvester ranged from 0 to 54 V, while those from the EM coil ranged from 0 to 10.6 V, resulting in a sum voltage of 10 to 60 V for the hybrid MME harvester. (b) Rectified voltage of the EM energy harvester and the sum of the two rectified voltages from the hybrid MME harvester at the 300 Ω impedance matching point of EM induction. (c) Rectified voltage of the piezoelectric MME harvester and the sum of the two rectified voltages from the hybrid MME harvester at the 30 kΩ impedance matching point of the piezoelectric MME harvester. (d) Calculated RMS power of the piezoelectric MME harvester, EM coil, and hybrid MME harvester under the load resistance ranging from 50 Ω to 500 kΩ.

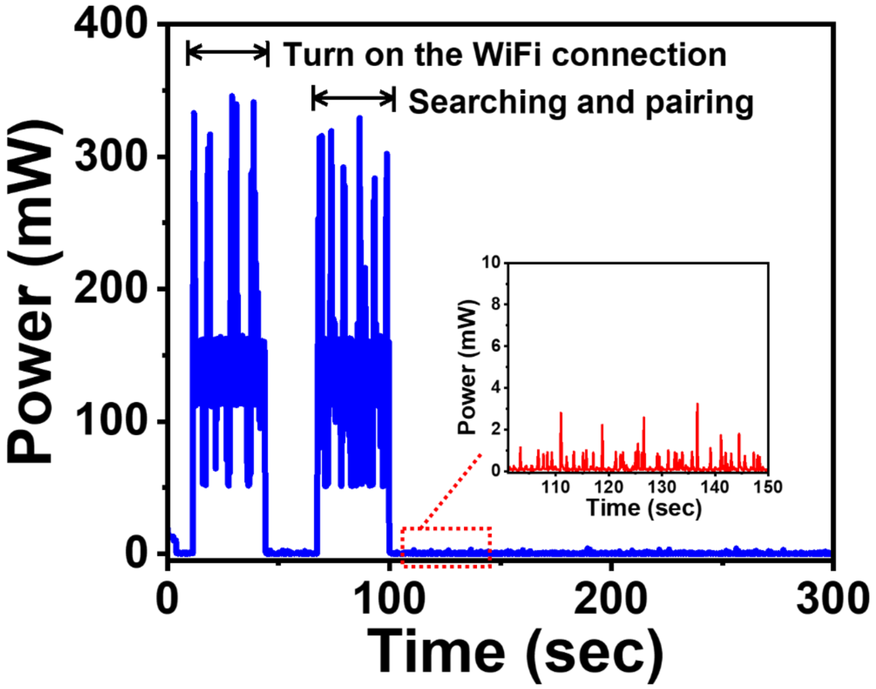

Figure S8. Power consumption of a Wi-Fi temperature and humidity sensor used in this study. When it was initially activated, the sensor consumed a maximum peak power of up to 335 mW for device start-up, even if it was instantaneous. During the process of pairing with a mobile device through Wi-Fi communication, the sensor required a peak power of approximately 310 mW. Subsequently, data were transmitted continuously every 10 s, with a monitored peak power consumption of approximately 3 mW, and the power demand significantly decreased compared with that during the awakening and pairing stage.

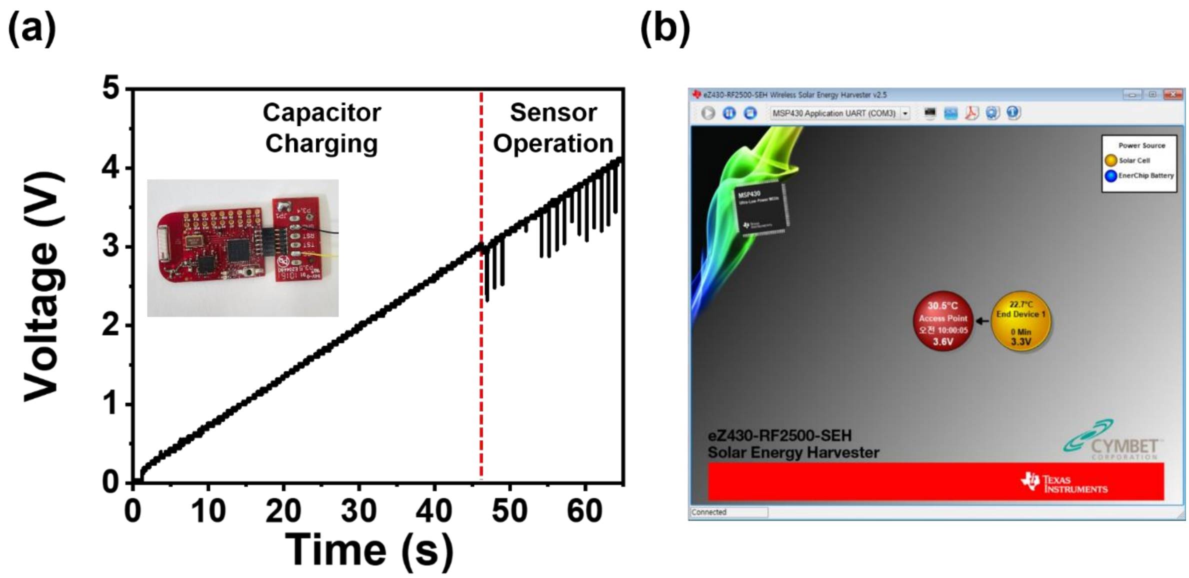

Figure S9. (a) Voltage curve of a 0.1 mF capacitor during energy storage by the hybrid MME generator and the subsequent operation of an IoT temperature sensor. (b) Screenshot of the personal computer monitoring system displaying temperature data transmitted from the IoT sensor.
